# Evaluation of Supplement Use in Sport Climbers at Different Climbing Levels

**DOI:** 10.3390/nu15010100

**Published:** 2022-12-25

**Authors:** Anna Chmielewska, Bożena Regulska-Ilow

**Affiliations:** Department of Dietetics and Bromatology, Wroclaw Medical University, 50-556 Wrocław, Poland

**Keywords:** sport climbing, dietary supplements, supplementation, sports nutrition

## Abstract

The lack of specific recommendations on the use of supplements for sport climbers may be the reason for their misuse by athletes of this discipline. This study aimed to evaluate choices of dietary supplementation, the reasons for taking them, and the source of information on supplementation among sport climbers at different levels. In addition, how climbers subjectively evaluated the impact of their diets in supporting selected aspects of climbing training was evaluated. We enrolled 110 regular sport climbers (40 women and 70 men) from Wroclaw, Poland, who completed a validated questionnaire, assessing their use of dietary supplements, attitudes towards the influence of diet on sports performance, and climbing level. Their anthropometric measurements were also collected. Participants regarded diet as an important element of sports performance. Sport climbers indicated the Internet to be the main source of information on supplements. Health maintenance and improvement of recovery were the most frequently chosen reasons for taking dietary supplements. The most common supplements were isolated protein, vitamin C, vitamin D, magnesium, and amino acid blends. However, participants rarely used supplements suggested as beneficial for sport climbing performance. Therefore, developing recommendations for supplementation in sport climbing and promoting this should be an elementary part of the preparation for climbing training.

## 1. Introduction

Dietary supplements are commercially available products that are consumed in addition to the regular diet. They include vitamins, minerals, plant extracts, amino acids, and many other substances [1]. Dietary supplements are defined as food, food components, or non-food compounds, which are purposefully ingested in addition to the normal diet to achieve a specific health or performance benefit [2]. The prevalence rates of supplement use among athletes are estimated to exceed 90%, depending on the sport or definition [3]. Some studies even suggest that supplement use may be as high as 100% in some team sports, with many athletes taking multiple daily supplements throughout the calendar year [4].

Supplements can have a place in an athlete’s diet [4,5]. Some are well-established performance enhancers, while others may augment both muscle and brain performance. Indeed, dietary supplements can be valuable for enhancing muscular adaptations to exercise, improving brain performance, decreasing delayed onset muscle soreness (DOMS) or pain, reducing injury severity, enhancing recovery from injury, reducing gastrointestinal problems, and decreasing illness load related to respiratory tract infections. As such, dietary supplements can help athletes to train and compete more effectively by reducing impediments to performance [5].

According to the 2018 International Olympic Committee (IOC) [2] and the 2019 International Amateur Athletics Federation (IAAF) [6], some dietary supplements can effectively support a sports nutrition plan, particularly if athletes fail to follow a balanced diet due to their lifestyle or if they have several consecutive competitions. The Australian Institute of Sport (AIS) created a regular sports supplement framework that recognizes the small but important role that sports foods and supplements play in the diet plans of high-performance athletes [7]. The framework was developed based on scientific evidence, safety, legality, and effectiveness in improving sports performance. It classifies nutritional supplements into four groups, A, B, C, and D, which are used to develop recommended best practice protocols for athletes [8]. However, supplements may contain undeclared doping substances that are banned by many sports associations. Contamination can occur during the manufacturing process, whilst banned substances can be intentionally not listed on the label or labeled under a different name [3]. 

Despite the purported benefits of supplements, they can have a negative impact on health and performance [3,9,10]. In addition to supplementation timing, the optimal dosage also needs to be considered. Furthermore, the uncontrolled use of supplements, herbal extracts, and medicines can cause many side effects [9]. Indeed, the intake of more than one supplement (polypharmacy) in high doses for long periods poses serious concerns regarding their safety. Kidney injury, gastrointestinal discomfort, hypercalcemia, and increased risk of cardiovascular diseases are only a few possible side effects of excessive supplement use [9]. Different studies suggest that 10–25% of European and American dietary supplements pose a potential risk of doping [3], with more recent reports claiming it to be as high as 38% [10].

Sport climbing is enjoying growing popularity, with bouldering and lead climbing the most frequently practiced disciplines [11]. Bouldering involves climbing up a short (usually 4–5 m) technical route, with mats on the ground for safety. Lead climbing is a discipline based on leading long routes (usually 20–40 m) with the use of rope and quickdraws clipped to bolts placed in the rock or an artificial wall [12]. It debuted as an Olympic discipline in 2020 [13], although this was postponed to the year 2021 due to the SARS-CoV-2 pandemic [14].

There are a growing number of studies focusing on supplement use in different populations of athletes [15,16,17]. A systematic review of the nutrition knowledge of athletes and coaches by Trakman et al. [15] reported many knowledge gaps in the field of supplement use, including incorrect beliefs regarding the use of ergogenic aids and the application of vitamin, mineral, and protein supplements. In addition, a study of a group of team sport players showed a low correlation between the subjective perception of the player’s knowledge in the field of supplementation and the objective evaluation of this knowledge, as assessed by the researchers. It was suggested that a lack of knowledge puts those athletes, who perceive their knowledge of dietary supplements to be high, in danger of inappropriate usage of dietary supplements. This leaves them vulnerable to the possible detrimental consequences of supplement misuse and highlights the importance of systematic and organized education on this topic [16]. However, the information on supplement use knowledge in sport climbing is lacking, and only a few studies have focused on the supplements used by climbers [18,19] or assessed which groups of supplements could enhance climbing performance [13]. Due to the lack of climbing-specific recommendations, sport climbers are adapting recommendations for other disciplines, which may not give the expected results.

The aim of the study was to evaluate dietary supplement use and types among sport climbers. We also aimed to assess how climbers subjectively perceived the impact of their diets on supporting selected aspects of climbing training. It was hypothesized that as the climbing level of the athletes increased, their knowledge about nutrition in different aspects of training performance should also increase. As such, climbers from the Elite group should perceive nutrition as more important in their training success than Intermediate and Advanced groups. Indeed, those in the Elite group may have a wider knowledge about discipline-specific supplement use due to the volume and intensity of their training. Based on this, we hypothesized that the frequency of choosing dietary supplements and their type would differ between groups representing different climbing levels. In addition, the study aimed to identify the reasons for taking dietary supplements and the sources of information on supplementation among sport climbers. 

## 2. Materials and Methods

### 2.1. Participants

The study included 110 regular sport climbers (40 women, 70 men) from Wroclaw, Poland. The criterion for inclusion in the study was at least one year of regular sport climbing. The exclusion criterion was recreational climbing with less than once-a-week climbing practice. Information on the study was spread through social media and directly through the trainers of the climbing gyms (5) at the time. All climbers who were willing to take part for the duration of the study (2019–2021, with an obligatory break during the COVID-19 pandemic) and who fulfilled the criteria were accepted. A survey was carried out in person by the participants during their visit. If they had any doubts, they could consult their answers, which ensured they were as accurate and reliable as possible. Participants gave their informed consent for the study protocol and were informed of the option to withdraw from the study at any time. The study was conducted in accordance with the Declaration of Helsinki and approved by the Institutional Ethics Committee of Wroclaw Medical University (number KB-45/2019).

### 2.2. Survey Tool

The questionnaire used in the study consisted of questions assessing the use of dietary supplements and had been previously validated in a group of Canadian athletes by Erdman et al. [20]. The questionnaire also included additional questions on climbing level and consisted of three parts. 

The first part contained ten questions on supplement use, including sources of knowledge about supplements, reasons for their use, places of purchase, and participants’ subjective assessment of how discontinuing supplements affected their climbing performance. Participants also listed all supplements they had taken in the preceding six months.

Another part of the questionnaire consisted of three questions and covered climbers’ dietary behaviors. This included questions on the type of diet they followed, a subjective evaluation of their diet, and an assessment of the importance of nutrition in supporting selected elements of climbing training. 

The last part of the questionnaire consisted of three questions and focused on participants climbing level and the frequency and duration of their training. Their climbing grade was determined by assessing three different routes established over the preceding six months. Participants were asked to indicate the highest grade, lead, or boulder they had managed to redpoint on three different routes/problems, on either an artificial wall or on a rock. The grade was then standardized according to the International Rock Climbing Research Association (IRCRA) scale. Participants were then stratified into a specific climbing level group, Intermediate, Advanced, or Elite, based on IRCRA scale score and gender [21]. For female climbers, Intermediate was a score between 10–14, Advanced 15–20, and Elite 21–26. For male climbers, Intermediate was a score between 10–17, Advanced 18–23, and Elite 24–27. 

### 2.3. Anthropometric Measures and Body Composition

Climbers’ weight and body composition was measured using an X-Contact bio impedance balance (Jawon Medical Inc., Seoul, Republic of Korea). Height was measured using a TANITA HR-001 stadiometer (TANITA Ltd., Tokyo, Japan). Participants were asked to come in the morning after an overnight fast. They were also asked not to perform intensive training in the evening before measurements. Based on the height and weight measured, the body mass index (BMI) of the participants was calculated.

#### Statistical Analyses

Patterns (frequencies) of the reasons for taking dietary supplements and the source of information on supplementation were recognized in the whole group. Differences in the frequency of using given supplements between the three defined climber groups (Intermediate, Advanced, and Elite) and between genders were analyzed with the use of Fisher’s exact test (when the expected size of a group was less than five) or Pearson’s χ^2^. The Cochran–Mantel–Haenszel test was used to assess the impact of belonging to a given level of climbers on gender differences. The effect of climbing level on the perceived importance of nutrition for supporting climbing performance was assessed using the Kruskal–Wallis test and differences between genders here were assessed using Mann–Whitney U test. Differences were recognized as statistically significant when *p* < 0.05. For statistically significant differences, a post hoc test was performed to assess differences between the study groups. Descriptive statistics were generated according to the anthropometric measurements of the study group (Table 1). Data analysis was performed with STATISTICA 13.3 (StatSoft Inc., Tulusa, OK, USA).

## 3. Results

All of the participants who were willing to take part and who fulfilled the inclusion criteria (*n* = 110), filled out the questionnaire and underwent anthropometric measurements. Table 1 shows the results of anthropometric measurements. Among the surveyed climbers, 46% declared that they followed an omnivorous diet and 30% limited their intake of animal products. A total of 25% of respondents declared that they followed a vegetarian diet, 3% declared following a vegan diet, and 4% of the participants declared following other diets, including the elimination diet.

Table 2 shows the percentage of the study group at each climbing level, the number of weekly workouts, and the time spent on climbing training per week. Climbers at the elite level declared more workouts and a higher volume of time spent on training units compared to the Advanced and Intermediate groups.

Table 3 shows the percentages of supplement types used over the past 6 months among the three different climbing levels in the female and male cohort.

The most common supplements were isolated protein supplements (whey protein concentrate or isolate, vegan concentrates from soy, and plant protein blends), vitamin C, vitamin D, magnesium, and amino acid blends (BCAAs or EAAs). Regarding supplements not included in the table, individual athletes declared taking supplements for joint regeneration (collagen and glutamine), plant extracts (berberine, ashwagandha and spirulina), probiotics, L-carnitine, beet juice, maca extract, and carbohydrate gels. The most frequent use of protein isolates, amino acids, vitamin C, creatine, magnesium, and multivitamin preparations was reported among elite climbers. However, there were no statistical differences between the caliber of athletes and the types of dietary supplements reported. The influence of gender on the use of supplements was borderline significant for creatine (*p* = 0.03, chi-square) and iron (0.045, Fisher’s test) when the whole groups of climbers were analyzed, and the Cochran–Mantel–Haenszel test indicates that belonging to a given level of climbers does not influenced these differences—the smallest *p* was greater than 0.05.

Table 4 presents the mean value for the indicator of the subjective assessment of the role of diet in supporting preparation for climbing training. Perceiving nutrition as an important factor influencing different training aspects may show the motivation for supplement use. This is particularly true when the diet is imbalanced or during an intense training period when it is difficult to maintain a proper intake of selected dietary components.

In addition to increased strength, the mean indicator for the subjective assessment of the role of diet in supporting selected aspects of climbing training was higher in the group of elite athletes compared to other groups. However, the difference was only statistically significant for recovery. Significant differences between genders were found for hydration in the Intermediate and Advanced groups.

Of the surveyed climbers, 77.3% declared using dietary supplements. This included 67.5% in the female group and 84.3% in the male group.

**Figure 1:**
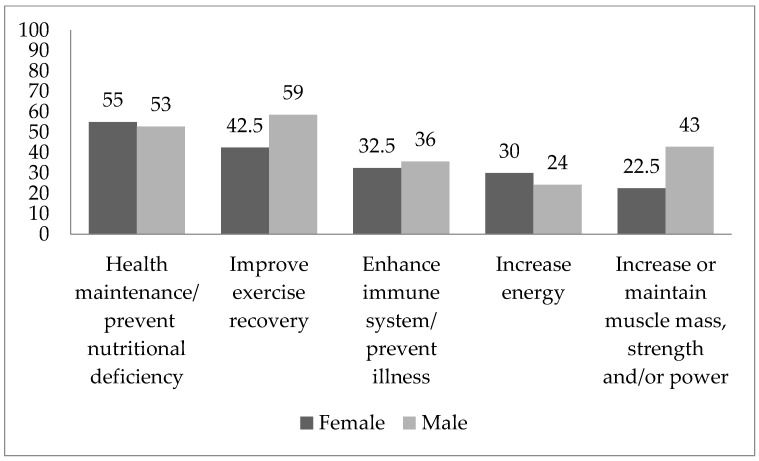
Comparison of rationale for taking dietary supplements in female and male group, presented as proportion of each cohort (%).

Figure 1 presents the reasons declared for taking dietary supplements among the surveyed climbers, according to the number of indications. The most often indicated reason was “Health maintenance/prevent nutritional deficiencies” followed by “Improve exercise recovery”. Male climbers more often indicated “Improve exercise recovery”, “Enhance immune system, prevent illness”, and “Increase or maintain muscle mass, strength and/or power” compared to female climbers, whereas representatives of the female group more often indicated “Health maintenance/prevent nutritional deficiencies”, “Increase energy” compared male climbers.

**Figure 2:**
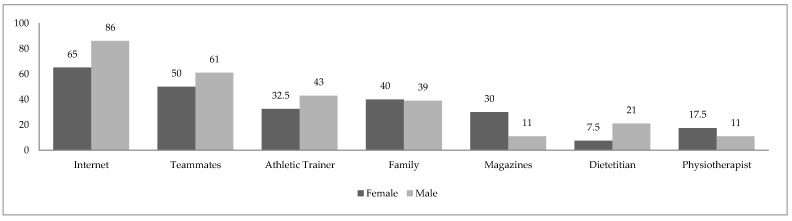
Comparison of most often indicated resource of dietary supplement information in female and male group, presented as proportion of each cohort (%).

Figure 2 shows declared sources of information on supplements. The most frequently cited sources were the Internet and other climbers, followed by family members and trainers. Male climbers more often indicated the Internet, teammates, and athletic trainer compared to female climbers, whereas representatives of the female group more often indicated family members, magazines, and physiotherapists compared male climbers. Other sources included books and specialized courses, as well as knowledge from studies. 

## 4. Discussion

This study aimed to evaluate the different aspects of supplement use among sport climbers, and their attitudes to proper diet as a component of sports performance. It was hypothesized that the use of dietary supplements would differ among the representatives of different climbing levels. The analysis of the results obtained showed a great lack of practical knowledge on the topic of supplement use, which was independent of the climbing level.

When developing nutrition plans to ensure an adequate intake of macronutrients and micronutrients, it is recommended to follow the “food first” approach, i.e., obtain nutrients from natural sources. However, it can be difficult or sometimes even impossible for diet alone to provide nutrients with established ergogenic or anabolic benefits. Moreover, obtaining some nutrients in sufficient quantities may require excessive caloric intake or consumption of undesirable nutrients [4].

Many sport climbers wish to have a low body mass and low body fat to enhance performance. Therefore, they may restrict calories, which can lead to inadequate macronutrient and micronutrient intake [22]. On the other hand, providing nutrients with ergogenic potential can result in energy surplus and unintended weight gain. In our study, climbers most commonly chose isolated protein supplements, amino acids, sports drinks, vitamin C, vitamin D, B vitamins, and magnesium. Energy and protein bars were also popular choices. According to the AIS, isolated protein supplements, vitamin D, sports drinks, and energy and protein bars belong to Group A supplements [6], with strong scientific evidence for their use in sports, according to evidence-based protocols. In our study, 48% of climbers declared limiting or eliminating animal products. Therefore, they used isolates to replace animal protein. The frequent use of protein supplements has also been found in other studies on athletes [23].

The supplementation of vitamin D at a dose of 800–2000 IU/day is recommended for the Polish population throughout the year [24]. In our study, around 30% of study participants supplemented vitamin D in each study group. Considering the above-mentioned recommendations, this seems to be a small percentage. Studies on Polish athletes showed that they had varying serum concentrations of 25-hydroxyvitamin D (25[OH]D) and that this was dependent on their discipline and their exposure to the sun during the summer and at winter training camps. Furthermore, athletes practicing indoor disciplines did not reach the recommended level of serum vitamin D all year round, even in the summer [25]. Heikkinen et al. also reported the low consumption of vitamin D among Finnish athletes [23]. Meanwhile, a study involving Dutch athletes showed that vitamin D was taken by significantly more athletes who received dietary counseling [26]. 

In the current study, climbers at intermediate and advanced levels consumed isotonic drinks more frequently compared to elite climbers. Sports drinks, according to the World Anti-Doping Agency (WADA), are in the same category as sports food [27] and are generally considered safe for use by athletes due to a reduced risk of contamination with banned substances. Sports drinks and bars provide an additional source of calories and are considered a convenient source of nutrients when it is impractical to consume everyday foods [6]. Furthermore, sports drinks are widely available in vending machines, grocery stores, and sports facilities, which may influence the prevalence of their consumption. It is worth noting that sports drinks typically contain carbohydrates, which are only recommended if training lasts for longer than 60 min [28]. Sport climbing training units often last longer than 60 min, however, their intensity is not continuous and is based on intervals. Therefore, the actual duration of physical effort can be much shorter. 

According to the AIS, vitamin C belongs to Group B supplements [6], which are considered for use by athletes under a case-managed monitoring situation. However, scientific evidence indicating their effectiveness needs to be further investigated. It is thought that vitamin C supplementation may be beneficial in reducing symptoms of upper respiratory tract infection (UTRI) in athletes [29]. In this regard, it has been estimated that a lower number of sick days is one of the factors differentiating medal-winning athletes in the World Championships and Olympic Games from other high-level international athletes [30]. Indeed, the duration of infection can significantly affect an athlete’s ability to participate in training during the year, and the results of research indicate that the number of sick days may determine success in Olympic-level competitions. The use of vitamin C in healthy individuals is considered safe. There is strong evidence that daily supplementation with 0.25–1.0 g/of vitamin C may lead to a lower frequency of URTI symptoms in athletes exposed to extreme physical stress, including marathon runners, skiers, and soldiers on subarctic operations [31]. Heikkinen et al. reported vitamin C intake in about 20% of the athletes they studied [23]. 

The AIS-classified BCAAs and magnesium as Group C supplements [6], which means that there is no scientific evidence supporting their benefit among athletes. Therefore, choosing supplements with limited evidence for their effectiveness may suggest that the study group has a low level of supplement knowledge and that they do not consult with a dietician or a doctor on their use. In other studies, BCAAs and magnesium were also among the most common supplements used by athletes [1,32]. 

Climbers may benefit from supplementing with beta-alanine [11,14], nitrates, sodium bicarbonate, and caffeine [11]. Some climbers may also benefit from creatine supplementation; however, side effects, such as fluid retention and weight gain, may negate any performance benefits [11]. All of these supplements were classified as Group A supplements by the IAS in 2014, which means that they have the potential to enhance athletic performance [6]. These supplements can improve climbing training effectiveness, and their inclusion in the diet can alleviate “pump” or lactate accumulation during rock climbing [11]. In our study, climbers declared supplementing beta-alanine and creatine, but they were not the most frequently chosen supplements. None of our study participants declared taking sodium bicarbonate or caffeine, and only one respondent declared drinking nitrate-rich beetroot juice. It can be assumed that climbers consumed caffeine in coffee or tea, or had it provided by another natural source. Peoples et al. also reported a very low intake of nitrate and bicarbonate in their study participants [19]. Natural sources of nitrates include beets and green leafy vegetables. A high intake of these products may partially ensure a high supply of nitrate and caffeine in the diet; however, due to the variability of nitrate and caffeine in products of natural origin, it is difficult to determine an athlete’s daily intake of these substances [33]. 

Our study participants declared supplementation with maca extract, which, according to the AIS, belongs to Group D supplements. The extract itself is not prohibited for use in sports; however, it may be contaminated with banned substances, and taking it as a dietary supplement is not recommended [6].

In summary, the choice of supplements used by the climbers studied was rather random. They were not only unrelated to the specific sports training they were undertaking, but it is also doubtful if they could prevent nutritional deficiency, which was the main reason declared for supplementing. Similar results were observed in a study on Spanish athletes, in whom the most prevalent group of substances chosen were those supported by little scientific evidence. It was suggested that this effect was produced by the availability of a high number of supplements on the market that contain minimal evidence for their effectiveness [15].

In studies conducted among different populations of athletes, authors have reported the frequent use of multivitamin preparations [34]. In our study, 12.7% of climbers declared taking multivitamin preparations. The choice of dietary supplements based on unreliable knowledge conveyed by the Internet and teammates, or based on recommendations for other sports disciplines, can lead to inadequate or unnecessary use of supplements with no proven impact of improvement in athletic performance [35].

A properly balanced diet should provide all essential nutrients, vitamins, and minerals at optimal levels to meet the requirements of the body [4]. Respectively, 20% and 4% of intermediate climbers followed a vegetarian or vegan diet. In the group of advanced climbers, 44% of respondents declared following a semi-vegetarian diet. Meanwhile, 75% of elite climbers were omnivores. In a study by Peoples et al. [9], an omnivorous diet was the most common across the groups (60% of Elite, 56% of Advanced, and 61% of Intermediate climbers), while less than 10% of the study participants declared that they followed a vegetarian or vegan diet.

There are little data on the prevalence of vitamin and mineral deficiencies among athletes. Furthermore, estimates generated for the general population are of little relevance to the population of professional athletes. Most nutritional surveys of athletes are based on dietary records or prospective data and are often compared with reference nutrient intakes (RNIs). This is of little value because the RNI is a population estimate, and, by definition, we expect that most of the population (98%) would have a nutrient requirement that is less than the RNI of the athletes. Deficiencies in an individual athlete are identified by measuring appropriate biomarkers [36].

Participants in the current study reported nutrition to be an important part of climbing training, which may be evidence of their high nutrition education and awareness of the impact of pre-and post-workout nutrition on selected aspects of training. The mean value on a scale of 1 to 5 (where 1 means not significant and 5 means very significant) was higher across all groups compared to the values declared by climbers in the study by Peoples et al. In our study, climbers declared nutrition was most important for “recovery”, followed by “body weight” and “hydration”. In contrast, in the study by Peoples et al., participants reported that nutrition was most important for “hydration” and least important for preventing DOMS [19]. 

In the current study, health maintenance and improved recovery were the main reasons for supplement use. One of the least popular reasons was medical indications, such as nutrient deficiency. Heikkinen et al. reported similar reasons for dietary supplementation among Finnish athletes [23]. The main reason for vitamin and mineral use was to prevent nutritional deficiencies and maintain health, and the main reason for dietary/nutritional supplements was recovery from exercise, as well as increased energy, muscle mass, power, and speed [23]. In a study involving Sri Lankan athletes, improved performance and improved overall health status were the most frequent reasons for taking supplements [37]. 

In our study, the most popular sources of information about supplements were online sources, followed by other climbers and family members. In a meta-analysis by Halabchi et al. [38], which evaluated supplementation consumption among Iranian athletes, the most prevalent source of information about supplement use were trainers and physicians, followed by teammates and dieticians. In a study by Baltazar-Martins et al. [15], conducted among elite Spanish athletes, most study participants got their information on supplement use at a store, followed by online sources and a sponsor. In our study, participants most frequently declared that they did not have any consultation regarding the choice of supplements used. A smaller percentage of study participants declared consulting a doctor or a nutritionist about the supplements they used.

## 5. Conclusions

No statistically significant differences were found in the use of supplements between the groups of climbers at different levels. Our study participants most commonly chose supplements with little scientific evidence for use in climbing. Additionally, they declared little or no use of supplements with proven effects, such as beta-alanine, nitrates, and sodium bicarbonate.

The representatives of female and male climbers indicated selected sources of knowledge about supplements, as well as reasons for using supplements with different frequency. 

The choice of non-climbing specific supplements that do not have scientific backing is probably influenced by the source of information most frequently chosen by both genders, the Internet. Therefore, proper education on where to source trusted information on the Internet would be an excellent move forward. This would allow climbers to make informed decisions and would encourage them to make responsible supplementation choices. Developing recommendations for supplementation in sport climbing and promoting this knowledge through appropriate nutrition education should be an elementary part of the preparation for climbing training.

## 6. Future Research

A limitation of the study is that data were collected from the population of sport climbers in only one city, Wrocław. It would be necessary to repeat the study protocol in other Polish cities to see if the observed tendencies represent the bigger scale of the issue. Comparing results to similar research led by representatives of sport climbers from other countries would also enrich the state of knowledge on the topic.

It would be interesting to evaluate the effectiveness of education on proper nutrition and responsible supplement use, as well as on the smart use of social media and the Internet as a source of information. This would allow for the identification of the most adequate ways of improving the knowledge of sport climbers, in relation to wise supplement choices.

## Figures and Tables

**Table 1:** Anthropometric measurements of the study group.

Measured Parameter	Female (*n* = 40)	Male (*n* = 70)
	x ± SD	x ± SD
Age (y)	30.13 ± 7.05	30.76 ± 6.17
Body mass (kg)	57.05 ± 649	72.2 ± 7.46
Heigh (cm)	166.1 ± 5.36	177.99 ± 6.26
BF (%)	21.79 ± 4.44	16.43 ± 4.49
BF (kg)	12.61 ± 3.63	12.57 ± 6.42
LBM (kg)	44.43 ± 4.06	60.16 ± 6.90

x—mean, SD—standard deviation, BF—body fat, LBM—lean body mass.

**Table 2:** Training frequency and duration per week depending on the climbing level and gender of the studied climbers, presented as the proportion of each cohort (%).

Training Parameter		Intermediate (*n* = 48)		Advanced *(n* = 50)		Elite (*n* = 12)	
		Female (*n* = 14; %)	Male (*n* = 34, %)	Female (*n* = 20; %)	Male (*n* = 30, %)	Female (*n* = 6; %)	Male (*n* = 6; %)
Training frequency	1–2/week	50	47	25	0	0	0
	3–5/week	50	53	70	93	67	83
	6–7/week	0	0	5	10	33	17
Hours of training per week	0–5 h/week	14	24	10	0	0	0
	6–10 h/week	64	56	50	33	17	17
	11–15 h/week	21	24	30	53	17	33
	16–20 h/week	0	0	10	13	50	33
	21–25 h/week	0	0	0	0	17	0
	>25 h/week	0	0	0	0	0	17

**Table 3:** Self-reported nutritional supplement and product use to support climbing performance in the last 6 months (proportion of each cohort (%)).

Supplement type	Intermediate (*n* = 48)		Advanced (*n* = 50)		Elite (*n* = 12)	
	Female (*n* = 14; %)	Male (*n* = 34; %)	Female (*n* = 20; %)	Male (*n* = 30; %)	Female (*n* = 6; %)	Male (*n* = 6; %)
B group vitamins	36	24	15	10	17	33
B-alanine	0	3	5	0	0	17
BCAA/EAA	7	15	20	20	17	17
Calcium	0	0	5	7	0	0
Creatine	7	18	5	20	0	33
Iron	14	0	5	0	0	0
Isotonic drink	14	29	15	23	0	17
Magnesium	29	18	25	17	17	50
Multivitamine	0	12	20	17	17	0
Omega 3 acids	7	12	15	3	0	17
Protein powder	43	41	35	57	67	83
Protein/energetic bars	14	18	35	10	0	17
Vitamin C	7	12	35	17	33	33
Vitamin D	21	32	25	20	33	17

**Table 4:** Comparison of the perceived importance (1 = not important, 5 = very important) of nutrition for supporting climbing performance. Data presented as mean (95% CI).

Training Aspect	Intermediate (*n* = 48)		Advanced (*n* = 50)		Elite (*n* = 12)		H-Value	*p*-Value
	Female (*n* = 14)	Male (*n* = 34)	Female (*n* = 20)	Male (*n* = 30)	Female (*n* = 6)	Male (*n* = 6)		
Preparation	4.07 (3.54–4.60)	3.97 (3.67–4.27)	4.25 (3.82–4.68)	3.93 (3.59–4.27)	4.67 (4.12–5.02)	4.17 (3.38–4.96)	2.110	0.348
Recovery	4.79 (4.53–5.03)	4.47(4.22–4.72)	4.65 (4.30–5.00)	4.33 (4.03–4.63)	5.00	5.00	6.478	0.039 **
Hydration	4.71 (444–4.98) *	3.91 (3.51–4.31)	4.75 (4.45–5.05) *	4.1 (3.72–4.48)	4.83 (4.40–5.26)	4.67 (3.81–5.52)	4.790	0.091
Body mass	4.36 (3.59–5.13)	4.26 (3.96–4.56)	4.65 (4.38–4.92)	4.53 (4.28–4.79)	4.83 (4.40–5.26)	4.50 (3.93–5.07)	2.433	0.296
DOMS	3.71(3.24–4.19)	3.21 (2.85–3.56)	3.60 (3.07–4.13)	3.23 (2.80–3.67)	3.33 (2.06–4.60)	3.83 (2.6–5.06)	0.469	0.791
Fatigue	3.93 (3.45–4.41)	3.79 (3.46–4.12)	3.85 (3.41–4.29)	3.77 (3.40–4.13)	4.17 (3.13–5.19)	4.50 (3.93–5.07)	3.415	0.181
Strength	4.29 (3.75–4.81)	4.18 (3.87–4.48)	4.25 (3.82–4.68)	4.00 (3.66–4.34)	4.17 (3.37–4.96)	4.17 (2.94–5.39)	0.270	0.874
Power	4.07 (3.59–4.55)	4.03 (3.70–4.36)	4.15 (3.69–4.61)	3.90 (3.58–4.22)	4.17 (3.37–4.96)	4.33 (3.48–5.19)	0.625	0.732
Endurance	3.86 (3.22–4.49)	3.65 (3.34–3.96)	4.05 (3.58–4.52)	3.73 (338–4.10)	3.83 (3.04–4.62)	4.50 (3.93–5.07)	1.826	0.401

* *p* < 0.05 versus male, ** *p* < 0.05 elite versus advanced.

## Data Availability

The data presented in this study are available on request from the corresponding author. The data are not publicly available due to privacy restrictions.

## References

[1] Knapik J.J., Steelman R.A., Hoedebecke S.S., Austin K.G., Farina E.K., Lieberman H.R. (2016). Prevalence of Dietary Supplement Use by Athletes: Systematic Review and Meta-Analysis. Sports Med..

[2] Maughan R.J., Burke L.M., Dvorak J., Larson-Meyer D.E., Peeling P., Phillips S.M., Rawson E.S., Walsh N.P., Garthe I., Geyer H. (2018). IOC consensus statement: Dietary supplements and the high-performance athlete. Br. J. Sports Med..

[3] Outram S., Stewart B. (2015). Doping Through Supplement Use: A Review of the Available Empirical Data. Int. J. Sport Nutr. Exerc. Metab..

[4] Close G.L., Kasper A.M., Walsh N.P., Maughan R.J. (2022). “Food first but not always food only”: Recommendations for using dietary supplements in sport. Int. J. Sport Nutr. Exerc. Metab..

[5] Rawson E.S., Miles M.P., Larson-Meyer D.E. (2018). Dietary Supplements for Health, Adaptation, and Recovery in Athletes. Int. J. Sport Nutr. Exerc. Metab..

[6] Burke L.M., Castell L.M., Casa D.J., Close G.L., Costa R.J.S., Desbrow B., Halson S.L., Lis D.M., Melin A.K., Peeling P. (2019). International Association of Athletics Federations Consensus Statement 2019: Nutrition for Athletics. Int. J. Sport Nutr. Exerc. Metab..

[7] The AIS Sport Supplement Framework. https://www.ais.gov.au/nutrition/supplements.

[8] Kim J. (2019). Nutritional Supplement for Athletic Performance: Based on Australian Institute of Sport Sports Supplement Framework. Exerc. Sci..

[9] Mohiuddin A.K. (2019). Supplements and enhancement drugs: Athletes torment themselves with potential risks. Int. J. Sport. Med. Rehabil..

[10] Duiven E., van Loon L.J., Spruijt L., Koert W., de Hon O.M. (2022). Undeclared Doping Substances are Highly Prevalent in Commercial Sports Nutrition Supplements. J. Sports Sci. Med..

[11] Michael M.K., Witard O.C., Joubert L. (2019). Physiological demands and nutritional considerations for Olympic-style competitive rock climbing. Cogent Med..

[12] Ginszt M., Goniewicz M., Ginszt A. (2012). Analiza przyczyn i następstw urazów u dzieci i młodzieży uprawiających wspinaczkę sportową. Hygeina Public Health.

[13] Lutter C., El-Sheikh Y., Schöffl I., Schöffl V. (2017). Sport climbing: Medical considerations for this new Olympic discipline. Br. J. Sports Med..

[14] Sas-Nowosielski K., Wyciślik J., Kaczka P. (2021). Beta-Alanine Supplementation and Sport Climbing Performance. Int. J. Environ. Res. Public Health.

[15] Baltazar-Martins G., de Souza D.B., Aguilar-Navarro M., Muñoz-Guerra J., Plata M.D.M., Del Coso J. (2019). Prevalence and patterns of dietary supplement use in elite Spanish athletes. J. Int. Soc. Sports Nutr..

[16] Trakman G.L., Forsyth A., Devlin B.L., Belski R. (2016). A Systematic Review of Athletes’ and Coaches’ Nutrition Knowledge and Reflections on the Quality of Current Nutrition Knowledge Measures. Nutrients.

[17] Sekulic D., Tahiraj E., Maric D., Olujic D., Bianco A., Zaletel P. (2019). What drives athletes toward dietary supplement use: Objective knowledge or self-perceived competence? Cross-sectional analysis of professional team-sport players from Southeastern Europe during the competitive season. J. Int. Soc. Sports Nutr..

[18] Gibson-Smith E., Storey R., Ranchordas M. (2020). Dietary Intake, Body Composition and Iron Status in Experienced and Elite Climbers. Front. Nutr..

[19] Peoples G.E., Scott Parker R.A., Craddock J. (2021). Rock climbers’ self-reported dietary practices and supplement use in the context of supporting climbing performance. J. Sport Exerc. Sci..

[20] Erdman K.A., Fung T.S., Doyle-Baker P.K., Verhoef M.J., Reimer R.A. (2007). Dietary supplementation of highperformance Canadian athletes by age and gender. Clin. J. Sport Med..

[21] Draper N., Giles D., Schöffl V., Fuss F.K., Watts P.B., Wolf P., Baláš J., Romero V.E., Gonzales G.B., Fryer S. (2015). Comparative grading scales, statistical analyses, climber descriptors and ability grouping: International Rock Climbing Research Association position statement. Sports Technol..

[22] Joubert L.M., Gonzalez G.B., Larson A.J. (2020). Prevalence of Disordered Eating Among International Sport Lead Rock Climbers. Front. Sports Act. Living.

[23] Heikkinen A., Alaranta A., Helenius I., Vasankari T. (2011). Dietary Supplementation Habits and Perceptions of Supplement Use Among Elite Finnish Athletes. Int. J. Sport Nutr. Exerc. Metab..

[24] Rusińska A., Płudowski P., Walczak M., Borszewska-Kornacka M.K., Bossowski A., Chlebna-Sokół D., Czech-Kowalska J., Dobrzańska A., Franek E., Helwich E. (2018). Vitamin D Supplementation Guidelines for General Population and Groups at Risk of Vitamin D Deficiency in Poland—Recommendations of the Polish Society of Pediatric Endocrinology and Diabetes and the Expert Panel With Participation of National Specialist Consultants and Representatives of Scientific Societies—2018 Update. Front. Endocrinol..

[25] Krzywanski J., Mikulski T., Krysztofiak H., Mlynczak M., Gaczynska E., Ziemba A. (2016). Seasonal Vitamin D Status in Polish Elite Athletes in Relation to Sun Exposure and Oral Supplementation. PLoS ONE.

[26] Wardenaar F.C., Ceelen I.J., van Dijk J.-W., Hangelbroek R., Van Roy L., Van Der Pouw B., De Vries J.H., Mensink M., Witkamp R. (2017). Nutritional Supplement Use by Dutch Elite and Sub-Elite Athletes: Does Receiving Dietary Counseling Make a Difference?. Int. J. Sport Nutr. Exerc. Metab..

[27] Raizel R., Coqueiro A.Y., Bonvini A., Tirapegui J., Grumezescu A.M., Holban A.M. (2019). Sports and Energy Drinks: Aspects to Consider. Sports and EnergyDrinks.

[28] Vitale K., Getzin A. (2019). Nutrition and Supplement Update for the Endurance Athlete: Review and Recommendations. Nutrients.

[29] Abioye A.I., Bromage S., Fawzi W. (2021). Effect of micronutrient supplements on influenza and other respiratory tract infections among adults: A systematic review and meta-analysis. BMJ Glob. Health.

[30] Schwellnus M., Soligard T., Alonso J.-M., Bahr R., Clarsen B., Dijkstra H.P., Gabbet T.J., Gleeson M., Hägglund M., Hutchinson M.R. (2016). How much is too much? (Part 2) International Olympic Committee consensus statement on load in sport and risk of illness. Br. J. Sports Med..

[31] Williams N.C., Killer S.C., Svendsen I.S., Jones A. (2019). Immune nutrition and exercise: Narrative review and practical recommendations. Eur. J. Sport Sci..

[32] Vento K.A., Wardenaar F.C. (2020). Third-Party Testing Nutritional Supplement Knowledge, Attitudes, and Use Among an NCAA I Collegiate Student-Athlete Population. Front. Sports Act. Living.

[33] Hord N.G., Tang Y., Bryan N.S. (2009). Food sources of nitrates and nitrites: The physiologic context for potential health benefits. Am. J. Clin. Nutr..

[34] Tscholl P., Alonso J.M., Dollé G., Junge A., Dvorak J. (2010). The Use of Drugs and Nutritional Supplements in Top-Level Track and Field Athletes. Am. J. Sports Med..

[35] Korczak R., Kruszewski M., Kruszewski A., Kuzmicki S., Olszewska A., Kepa G., Landowski K. (2016). Preferences in the use of nutritional supplements and the correctness of their selection for training purposes. Balt. J. Health Phys. Act..

[36] Maughan R.J., Shirreffs S.M., Vernec A. (2018). Making decisions about supplement use. Int. J. Sport Nutr. Exerc. Metab..

[37] Halabchi F., Shab Bidar S., Selk Ghaffari M. (2021). Prevalence of supplement consumption in Iranian athletes: A systematic review and meta analysis. Int. J. Prev. Med..

[38] De Silva A., Samarasinghe Y., Senanayake D., Lanerolle P. (2010). Dietary Supplement Intake in National-Level Sri Lankan Athletes. Int. J. Sport Nutr. Exerc. Metab..

